# Bcl-2 confers survival in cisplatin treated cervical cancer cells: circumventing cisplatin dose-dependent toxicity and resistance

**DOI:** 10.1186/s12967-015-0689-4

**Published:** 2015-10-16

**Authors:** Gina Leisching, Benjamin Loos, Matthys Botha, Anna-Mart Engelbrecht

**Affiliations:** Department of Physiological Sciences, Stellenbosch University, c/o Merriman and Bosman road, Stellenbosch, South Africa; Department of Obstetrics and Gynaecology, Stellenbosch University, Stellenbosch, South Africa

**Keywords:** Bcl-2, Beclin-1, Cisplatin, Cervical cancer, Apoptosis

## Abstract

**Background:**

Cisplatin is the main chemotherapeutic drug for the treatment of cervical cancers, however resistance to cisplatin is increasingly common and therefore has limited the efficacy and use of this drug in the clinic. Dose-dependent toxicity poses an additional challenge since patients suffer long-term and often permanent side-effects after treatment. Bcl-2 up-regulation has been implicated in the resistance to cisplatin in a variety of cancer cell lines, however its role in cervical cancer is confounding.

**Methods:**

A low, non-cytotoxic concentration of cisplatin was used in the treatment of HeLa and CaSki cells. Bcl-2 expression was determined through Western blotting and immunocytochemistry before and after treatment with cisplatin. To assess the reliance of the cervical cancer cells on Bcl-2 in the presence of cisplatin, Bcl-2 knock-down was achieved through RNA interference, where after apoptosis was assessed through PARP cleavage (Western blotting), Caspase activity (Caspase-Glo^©^) and PI inclusion analysis (Flow cytometry). Finally, pre-malignant and malignant cervical tissue was analysed for the presence of Bcl-2 through Western blotting and immunofluorescence.

**Results:**

Cervical cancer cells upregulate Bcl-2 when treated with a non-cytotoxic concentration of cisplatin, which when silenced, effectively enhanced cisplatin sensitivity, and therefore significantly induced apoptosis. Analysis of the expression profile of Bcl-2 in cervical tissue revealed its up-regulation in cervical carcinoma, which agrees with results obtained from the in vitro data.

**Conclusions:**

Our data strongly suggest that utilising a lower dose of cisplatin is feasible when combined with Bcl-2 silencing as an adjuvant treatment, thereby improving both the dose-dependent toxicity, as well as cervical cancer resistance.

## Background

It has been over 40 years since the clinical development of *cis*-diaminedichloroplatinum (II) (cisplatin). It has been considered as one of the most effective anticancer drugs against cervical cancer in neoadjuvant and salvage treatment [1]. Despite this success, resistance to cisplatin is increasingly common and often culminates in chemotherapeutic failure. It is suggested that cisplatin resistance, i.e. failure of cancer cells to undergo apoptosis, may be due to a disruption in the normal apoptotic response. Increases in cisplatin dosage as a means of circumventing this resistance has proven impractical since patients experience dose-dependent toxicity and long-term damage such as nephro- [2, 3] and ototoxicity [4–6], which further decreases the quality of life [7, 8]. It is therefore necessary to address both the issues of cisplatin dosage, as well as possible mechanisms of resistance in cisplatin treated cells. This may provide new avenues for chemotherapy and adjuvant treatments that would not only favour the use of lower concentrations of cisplatin, thereby limiting the chemotherapeutic side effects, but also lessen the potential for chemoresistance.

A systematic survey and meta-analysis of the transcriptional profiles of a variety of cancers indicated that the dysregulation of Bcl-2 is a key distinguishing factor between normal and cancer cells [9]; moreover, its increased expression has been correlated with increased resistance of a variety of cancers to chemotherapy drugs, including cisplatin [10, 11]. Evidence documenting the expression of Bcl-2 in cervical cancers is confounding, and the role of Bcl-2 under stressful conditions, such as chemotherapy treatment in cervical cancer cells remains poorly understood. It is therefore necessary to dissect the role of this anti-apoptotic protein in cisplatin resistance in cervical cancer.

Macroautophagy (hereafter referred to as autophagy) is in the majority of cases, considered to serve as a pro-survival mechanism, and is up-regulated under conditions such as nutrient depletion, hypoxia and chemotherapy treatment [12–14]. Apoptosis and autophagy share regulatory mechanisms [15] which play important roles, not only under normal physiological conditions, but also in disease states such as cancer. The interaction between Bcl-2 and Beclin-1 proteins is an important point of convergence between the apoptotic and autophagic pathways. The importance of this interaction is underlined by the fact that Beclin-1 is a tumour suppressor protein and that inhibition of its function by Bcl-2 contributes to the oncogenic potential of Bcl-2. Its expression often occurs in the absence of Beclin-1 in malignant cells, therefore ratio analysis of Beclin-1 and Bcl-2 expression as means of detecting the role of autophagy under conditions of apoptosis may have therapeutic value, such that it may be indicative of chemotherapeutic success of a treatment.

In the present study we address the issue of cisplatin dose-dependent toxicity by using a non-toxic concentration of the drug on two cervical cancer cell lines throughout. With this in mind, we aimed to (1) evaluate the basal levels of Bcl-2 and Beclin-1 in HeLa and CaSki cell lines, (2) silence Bcl-2 as a means of defining its role during cisplatin treatment, and finally analyse pre-malignant low-grade squamous intraepithelial lesions (LSILs), high-grade squamous intraepithelial lesions (HSILs), as well as malignant cervical tissue for the presence of Bcl-2 under basal conditions in order to evaluate its possible role in vivo during the progression of cervical cancer and validate the in vitro results.

## Methods

### Cell culture lines

HeLa and CaSki cells were purchased from Highveld Biological (Johannesburg, South Africa) and grown in Dulbecco’s Modified Eagles Medium (DMEM) supplemented with 10 % foetal bovine serum (FBS) (Gibco. Ltd) and 1 % penicillin/streptomycin (P/S) (Sigma-Aldrich, Johannesburg, South Africa). Cells were grown at 37 °C and 5 % CO_2_ under humidified conditions and passaged upon reaching 70–80 % confluency.

### Cisplatin treatment

Cisplatin (Sigma-Aldrich, Johannesburg, South Africa) was prepared before each treatment period by dissolving the powder in 0.9 % NaCl solution to obtain a 0.001 M stock solution. It was then added to sub-confluent cells to reach final working concentration of 15 µM and incubated for a period of 24 h. Cisplatin dose–response curves and non-toxic validation of the above mentioned working concentration is described in previous work published by our group [16].

### Immunocytochemistry

HeLa and CaSki cells were grown on coverslips in six-well plates (250,000 cells/well). Following the treatment period, the medium was removed and cells were washed once with PBS. Cells were fixed and permeabilised with ice-cold methanol and acetone (1:1) and left to incubate at 4 °C for 10 min. The fixative was then removed and coverslips were allowed to air-dry for a further 20 min where after they were rinsed twice with PBS. Non-specific binding was prevented by incubating cells with 10 % donkey serum for 1 h at RT. After this time period, the donkey serum was blotted off and Beclin-1 and Bcl-2 primary antibodies (Cell Signaling, MA, USA) diluted in 1 % bovine serum albumin (BSA, 1:50) were added to cells and allowed to incubate overnight at 4 °C. Cells were then rinsed three times with PBS and allowed to incubate with the appropriate secondary antibody (FITC donkey anti-rabbit and TxRed goat anti-rabbit, Cell Signaling, MA, USA) for 1 h at RT. 10 min before the completion of incubation, Hoechst 33342 (1:200) was additionally added for the remainder of the incubation period. Next, cells were rinsed and the coverslips were mounted on glass slides with DAKO fluorescent mounting medium (DAKO Inc., CA, USA). Slides were kept at −20 °C until analysis.

### Western blotting and immunofluorescence

Cells were lysed with RIPA buffer and whole protein extracts were analysed through Western blotting [17] and then incubated with the following primary antibodies (1:1000 dilution): Bcl-2, Beclin-1, cleaved PARP, LC-3II, p62 and β-actin (Cell Signaling, MA, USA). For immunofluorescence, a primary antibody recognising Bcl-2 (Cell Signaling, MA, USA) and a secondary antibody conjugated to a fluorophore (FITC donkey anti-rabbit, Cell Signaling, MA, USA) were used. Bcl-2 expression was assessed in all paraffin-embeded cervical tissue and representative images were acquired.

### siRNA transfections

Bcl-2 and control/scrambled siRNA were purchased from Cell Signaling Technologies (Beverly, MA, USA) and supplied as a 10 µM stock solution. The relevant proteins were silenced through reverse transfection. All transfections were performed using FuGENE6 (Roche, Johannesburg, South Africa) according to the manufacturers instructions. Cells were then incubated for 48 h before continuing with treatments and analyses. Silencing was confirmed through Western blotting to detect changes in total amount of the targeted proteins.

### Caspase 3/7 activity assay

The Caspase-Glo^®^ assay was purchased from Promega (Southampton, UK) and was performed according to the manufacturer’s instructions.

### Flow cytometry: propidium iodide staining

Cells were grown in T25 flasks at a seeding density of 700,000 cells/flask. After the treatment period, 2 ml Tryple-Xpress trypsin (Gibco) was added to each flask for 3–4 min until all cells had detached. The cell suspension was then added to 15 ml Falcon tubes and centrifuged at 6000×*g* for 3 min. The supernatant was removed and the pellet washed with 0.1 M PBS. The cells were centrifuged again at the same specifications and the supernatant was removed as before. PI (Sigma-Aldrich, South Africa) was added to the unfixed cells to obtain a final concentration of 1 mg/ml, incubated for 10 min and analysed on the flow cytometer (BD FACSAria I). A minimum of 10,000 events were collected and analysed using a 488 nm laser and 610LP, 616/23BP emission filters. PI inclusion signified loss in membrane integrity and cell death. Values were represented as a percentage of the control.

### Patients and specimen collection

The study protocol has been approved by the Research Ethics Committee of Stellenbosch University. Tissue collection was in accordance with the ethical standards of the responsible committee on human experimentation and with the Helsinki Declaration of 1975, as revised in 1983 (reference number: N09/02/045). Biopsies were collected from patients undergoing routine colposcopy screenings and hysterectomies at Tygerberg Hospital, Tygerberg, Western Cape. Samples were rinsed with PBS, placed in cryovials and stored individually in liquid nitrogen until further use. A total of 10 non-cancerous, 29 LSILs, 33 HSILs and 13 carcinoma biopsies were collected for analysis.

## Results

### Bcl-2 protein expression levels increase after cisplatin treatment in HeLa and CaSki cells

**Fig. 1:**
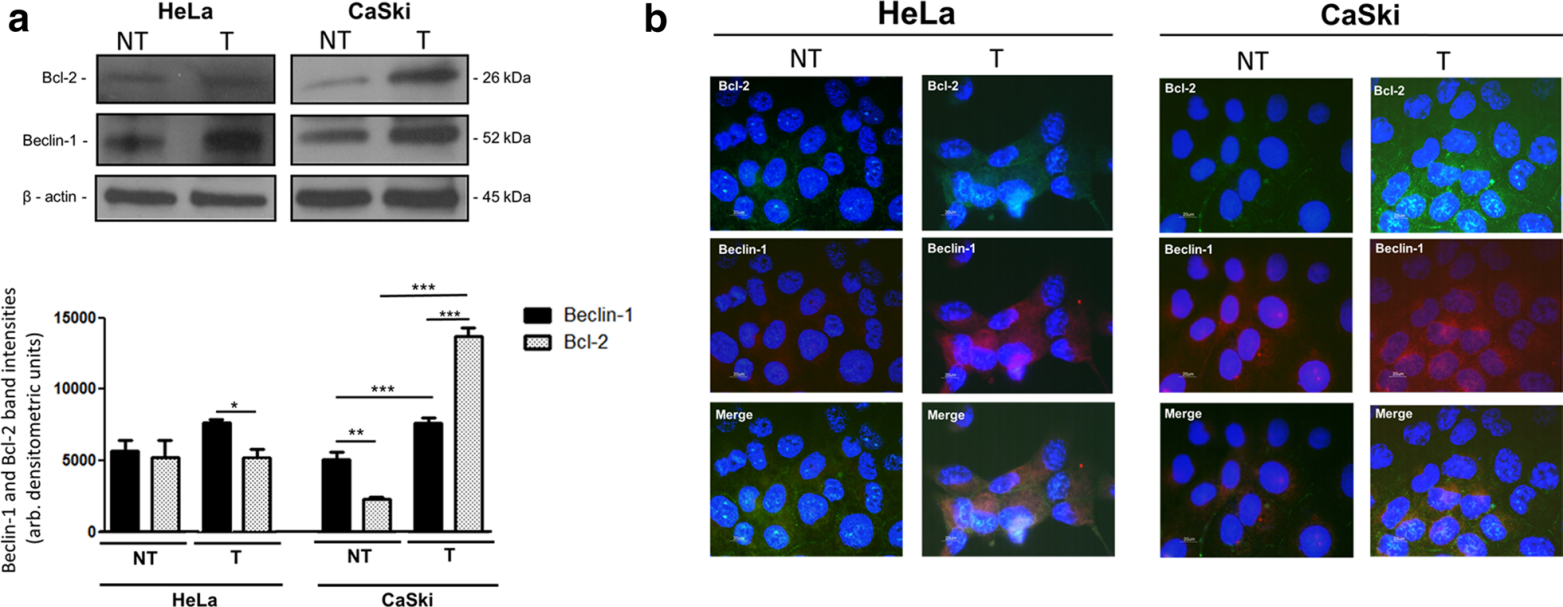
Analysis of Beclin-1 and Bcl-2 protein levels in non- treated (NT) and treated (T) HeLa and CaSki cells. **a** Bcl-2 and Beclin-1 protein levels in HeLa and CaSki cells were analysed for changes in expression after the addition of 15 μM cisplatin for a period of 24 h. *p < 0.05, **p < 0.01, ***p < 0.001, n = 3. Analyzed by one-way ANOVA with Bonferonni post hoc test. **b** Representative immunofluorescent images of HeLa and CaSki cells depicting expression of Bcl-2 and Beclin-1 under basal conditions (NT) and in response to cisplatin treatment (T). *Blue* Hoechst 33342, *green* Bcl-2 and *red* Beclin-1. *Scale bar* 20 μm, n = 3

Bcl-2 and Beclin-1 protein expression levels were analysed before (NT) and after (T) treatment with a non-toxic concentration of cisplatin for a period of 24 h (Fig. 1a). Following treatment with cisplatin, Bcl-2 protein levels increased significantly in HeLa cells. The addition of cisplatin (T) induced a significant increase in Beclin-1 and Bcl-2 protein levels in CaSki cells. Immunocytochemistry of both cell lines under treatment conditions confirm Western blotting data since treatment with cisplatin increases the fluorescent signal of Bcl-2 in both Hela and CaSki cells (Fig. 1b).

### Bcl-2 silencing increases intracellular Beclin-1 protein levels in HeLa and CaSki cells

**Fig. 2:**
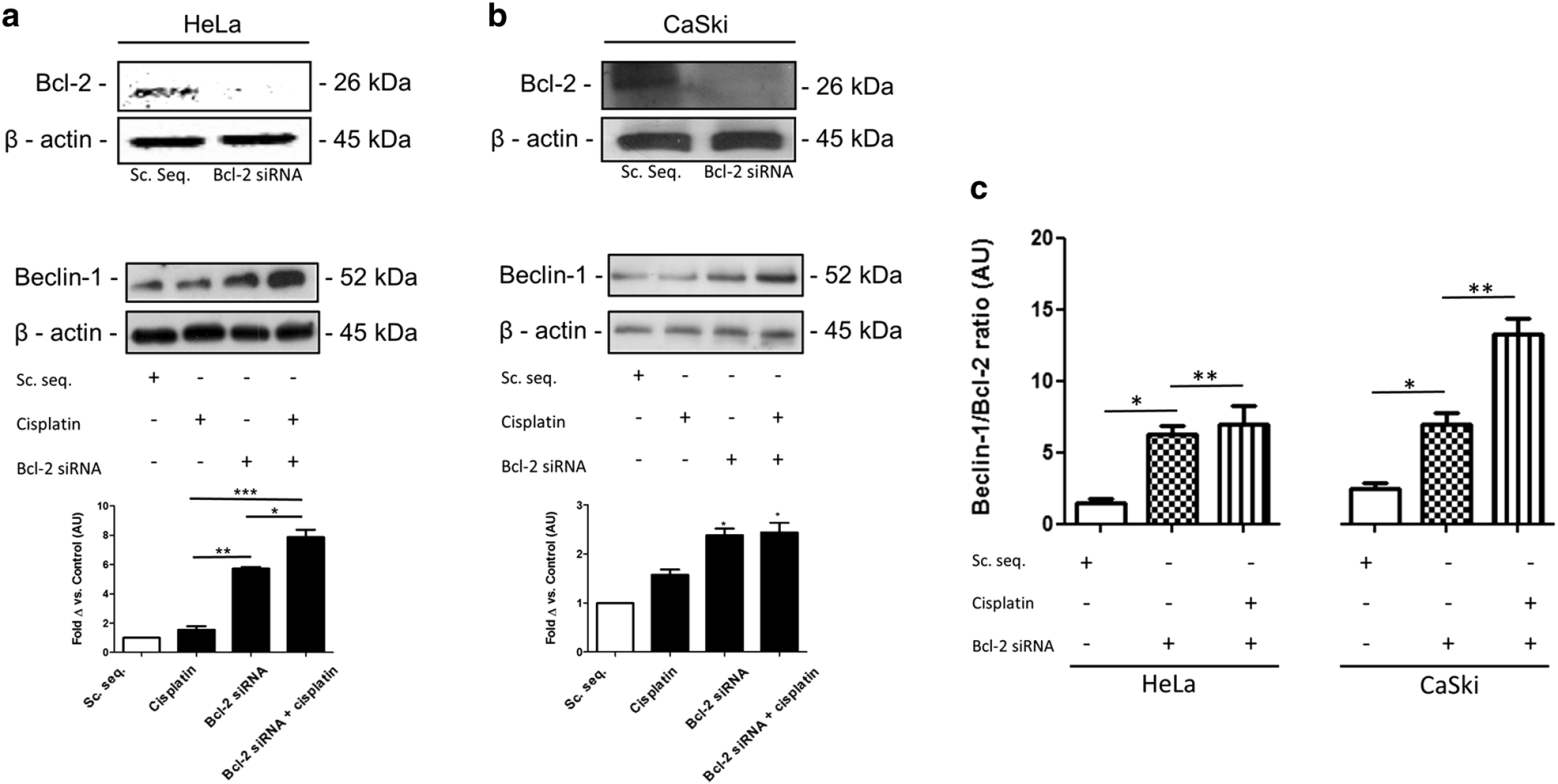
Silencing of Bcl-2 in HeLa and CaSki cells and resulting expression levels of Beclin-1. **a** Silencing of Bcl-2 confirmed through Western blotting. Beclin-1 protein expression in HeLa cells, *p < 0.05, **p < 0.01, ***p < 0.001, n = 3. **b** Silencing of Bcl-2 confirmed through Western blotting. Beclin-1 protein expression in CaSki cells, *p < 0.05 vs. cisplatin, n = 3. **c** Ratios were determined according to densitometric analysis of Beclin-1 and Bcl-2. *p < 0.05 and **p < 0.01, n = 3. *Sc. Seq.* scrambled sequence (control), *AU* arbitrary units

Silencing of Bcl-2 was performed in order to assess whether Bcl-2 modulates Beclin-1 expression under conditions of stress, such as during cisplatin treatment. Bcl-2 silencing was confirmed through Western blotting in both HeLa and CaSki cells (Fig. 2a, b). Beclin-1 protein levels increased significantly in both groups where Bcl-2 was silenced. The Beclin-1/Bcl-2 ratio was then assessed after silencing of Bcl-2 in HeLa and CaSki cells in order to determine whether a shift toward autophagic cell death has occurred, since Bcl-2 exerts an inhibitory effect on Beclin-1(Fig. 2c). The ratio increased significantly in both cell lines which is indicative of an autophagy dominant state within the cell.

### Bcl-2 silencing improves cytotoxicity of cisplatin and induces apoptosis in cervical cancer cells

**Fig. 3:**
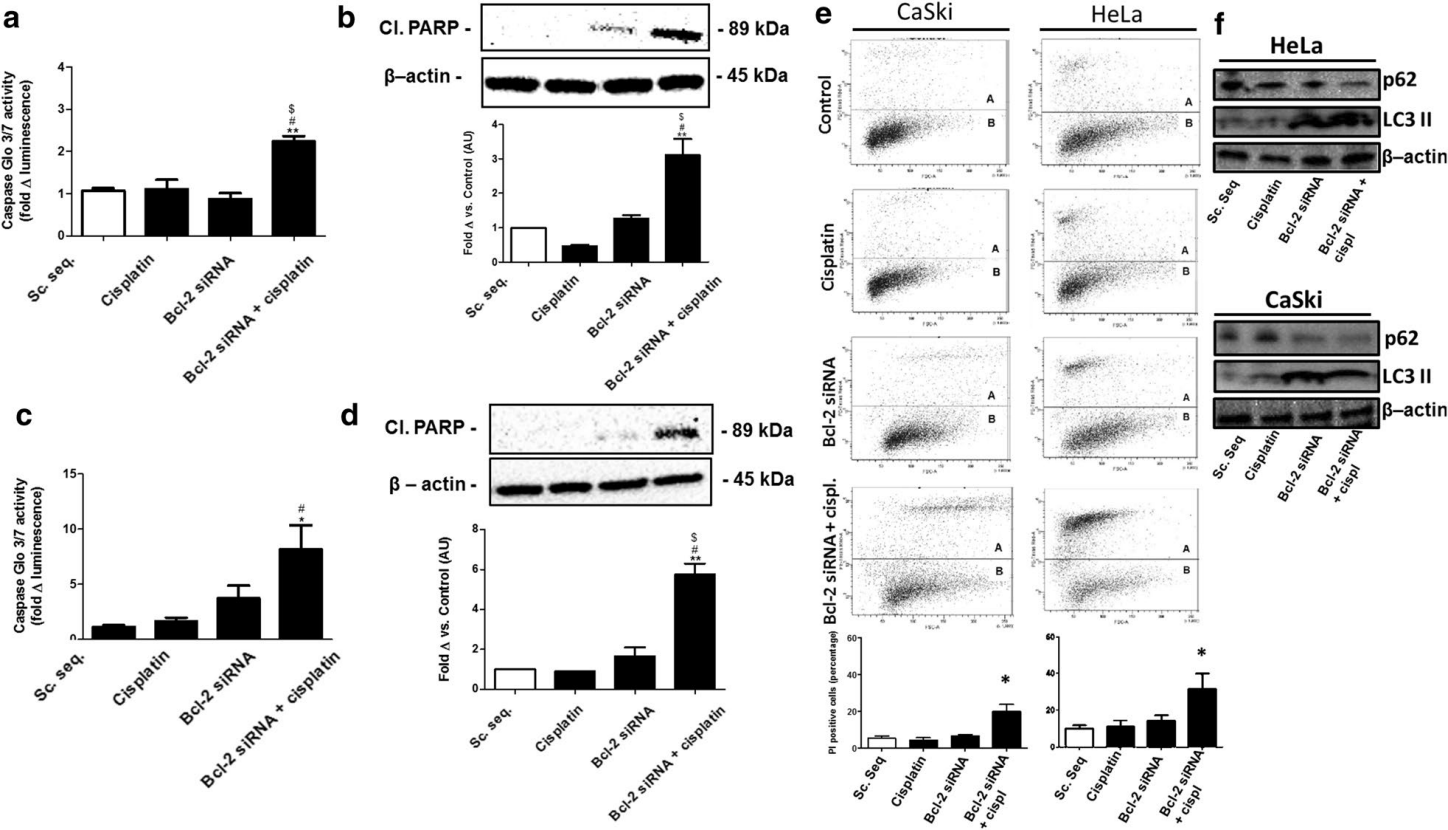
The effect of Bcl-2 silencing on apoptosis during cisplatin treatment in HeLa (**a** and **b**) and CaSki (**c** and **d**) cells. **a** Caspase -3/-7 activity, **p < 0.01 vs. sc. seq. ^#^ *p* < 0.01 vs. cisplatin and ^$^ *p* < 0.01 vs. Bcl-2 siRNA, n = 4. **b** PARP cleavage levels, **p < 0.01 vs. sc. seq., ^#^ *p* < 0.001 vs. cisplatin and ^$^ *p* < 0.01 vs. Bcl-2 siRNA, n = 3. **c** Caspase-3/-7, *p < 0.05 vs. sc.seq. and ^#^ *p* < 0.05 vs. cisplatin, n = 3. **d** PARP cleavage, **p < 0.01 vs. sc.seq., ^#^ *p* < 0.01 vs. cisplatin and ^$^ *p* < 0.01 vs. Bcl-2 siRNA, n = 3. **e** PI inclusion (Flow cytometry), PI positive: quadrant A, PI negative: quadrant B *p < 0.05 vs. Cont., Cispl. and Bcl-2 siRNA, n = 3. **f** Western blot detecting p62 and LC-3 II in HeLa and CaSki cells. *Sc. seq.* scrambled sequence (control), *AU* arbitrary units

Effector caspase activity, cleaved PARP and cell membrane integrity was analysed as a markers of apoptosis and cell death. Silencing of Bcl-2 significantly increased cisplatin-induced caspase activation in HeLa and CaSki cells (Fig. 3a, c). Equally, the silencing of Bcl-2 significantly increased cisplatin-induced PARP cleavage in these cells (Fig. 3b, d). The combination of Bcl-2 silencing and cisplatin treatment significantly increased the number of cells positive for PI staining as observed by flow cytometry (Fig. 3e). We also observed that Bcl-2 silencing alone and in combination with cisplatin decreases p62 protein expression and increases LC3-II expression (Fig. 3f), thus increasing autophagy.

### Bcl-2 protein expression is increased in cancerous cervical tissue

**Fig. 4:**
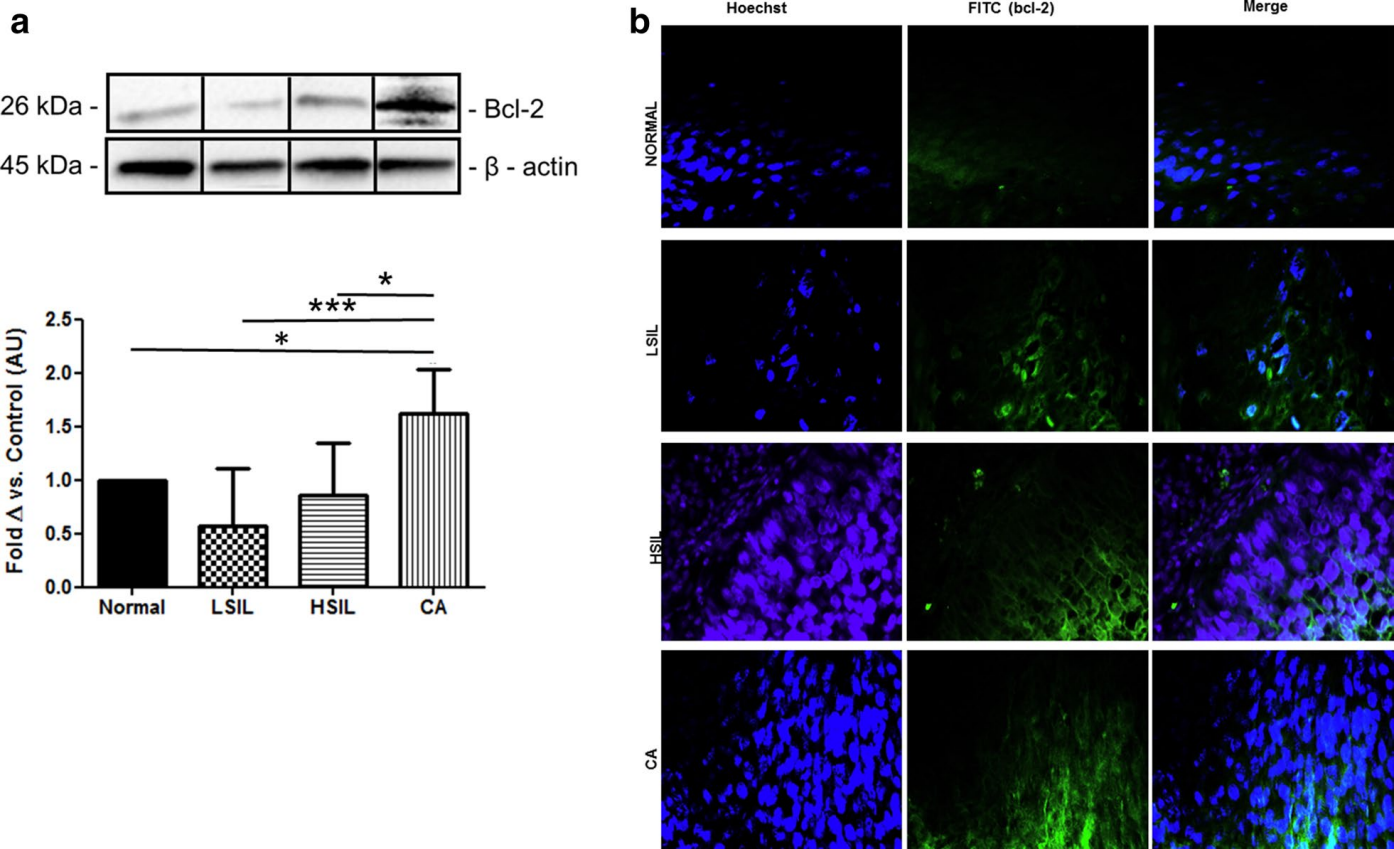
The Bcl-2 expression profile in normal, low-grade and high-grade squamous intraepithelial lesions and cervical carcinoma tissue of the cervix. **a** Representative Western blot, *p < 0.05, **p < 0.01, ***p < 0.001. Statistical analysis: ANOVA and Bonferroni correction. All results expressed as mean ± SEM, normal (n = 4), LSIL (n = 6), HSIL (n = 8) and cervical carcinoma (n = 5). **b** Representative fluorescent micrographs of Bcl-2 expression profiles in normal, LSIL, HSIL and cervical carcinoma, *scale bar* 20 μm. *LSIL* low-grade cervical intraepithelial lesions, *HSIL* high-grade squamous intraepithelial lesions, *CA* carcinoma

Bcl-2 expression was significantly increased in cervical carcinoma tissue in comparison to normal tissue, LSIL tissue and HSIL tissue (Fig. 4a). Fluorescent micrographs displayed an increase in Bcl-2 expression levels in carcinoma tissue exhibiting 95 % positivity in comparison to the control tissue (1 % positivity), LSILs (0 % positivity) and HSILs (2 % positivity) (Fig. 4b). All positive samples stained moderately to strongly with Bcl-2.

## Discussion

Cisplatin has been widely used to treat solid tumours and much success has been achieved from the use of this drug in the treatment of head and neck, ovarian, testicular, small-cell lung cancer and cervical cancers. The success of cisplatin treatment is limited due to its dose-limiting toxicity and the resulting, and often permanent side-effects. Equally challenging is the tendency of cancer cells to acquire resistance to cisplatin treatment. There is therefore a need for effective treatment options which utilise lower doses of cisplatin that are able to maintain cytotoxicity and address the issue of resistance.

Bcl-2 expression has been implicated in the resistance of various cancers to chemotherapy treatment, but evidence of its role in cervical cancer remains unclear. We therefore aimed to dissect the role of this oncogenic protein in two cervical cancer cell lines and in pre-malignant and malignant cervical tissue. Bcl-2 and Beclin-1 protein expression levels were analysed in HeLa and CaSki cells under control conditions (NT), as well as after a 24 h treatment period with a non-cytotoxic concentration of cisplatin (T) (Fig. 1a, b). Cisplatin treatment significantly increased Bcl-2 protein levels in both cervical cancer cell lines which was confirmed through immunocytochemistry. It is important to note that the cervical cancer cells were viable and had not undergone apoptosis at this point. We hypothesise that the upregulation of Bcl-2 that is observed may be responsible for the observed survival.

The upregulation of Bcl-2 in the presence of cisplatin prompted further analysis in order to elucidate its role in cervical cancer survival and its potential interaction with Beclin-1. Since Bcl-2 is known to associate with Beclin-1, an interaction that serves as a rheostat between apoptosis and autophagy induction, it was necessary to dissect whether this association was significant under treatment conditions. Silencing of Bcl-2 upregulated Beclin-1 expression in both cervical cancer cell lines. This observation is in accordance with a study that showed Bcl-2 silenced MCF-7 breast cancer cells increased Beclin-1 expression, and after 72 h resulted in autophagic cell death [18]. The Beclin-1/Bcl-2 ratio increased significantly in both cell lines with and without the treatment of cisplatin. Interpretation of these results allow a distinction to be made between autophagy acting as a survival mechanism and autophagy serving as a mechanism of cell death. Beclin-1 expression is required to be elevated for the latter to occur [19], which was observed in our model.

Apoptosis occurred in both cell lines after Bcl-2 was silenced. Analysis of apoptotic death markers suggests a role of Bcl-2 as a mechanism which delays the onset of apoptosis, thereby conferring resistance to cisplatin treatment. More importantly however, is the observation that the cytotoxicity of cisplatin improved greatly. Both HeLa and CaSki cells displayed increased caspase -3/-7 activity in response to the combination of Bcl-2 silencing and cisplatin treatment (Fig. 3a, c). Equally indicative of cell death induction was a significant increase in PARP cleavage in both cell lines (Fig. 3b, d). Here we show that a previously non-cytotoxic concentration of cisplatin becomes toxic to cervical cancer cells and provides conceptual evidence to suggest that lower dosages of cisplatin may be a viable approach to treatment when combined with Bcl-2 silencing.

The Beclin-1/Bcl-2 ratio after silencing (Fig. 2c), as well as various cell death (Fig. 3a–e) and autophagy markers (Fig. 3. F) indicates that cell death *with* autophagy [20, 21] may be occurring in this in vitro model, as the presence of apoptosis and autophagy is evident. Our findings agree with others in that the silencing of Bcl-2 improved sensitivity to cisplatin treatment in bladder cancer cells [22], melanoma cells [23], ovarian cancer cells [24], non-small lung cancer cells [25] and lung adenocarcinoma cells [26]. It is important to note that the above studies utilised concentrations of cisplatin which induced 50 % cell death or more. Here we use a concentration of cisplatin which does not induce cell death when administered as a single agent. This approach simultaneously addresses the challenge of cisplatin toxicity and chemotherapy resistance.

To confirm the relevance of the in vitro data, basal levels of Bcl-2 in cervical tissue was analysed. Analysis of cervical pre-malignant and malignant tissue revealed that Bcl-2 protein expression was significantly increased in cervical carcinoma tissue samples (Fig. 4a, b) and suggests that the malignant state requires such an alteration in order to maintain the malignant phenotype. An increase in Bcl-2 protein expression as demonstrated in this study is in accordance with the results of Dimitrakakis and colleagues where it was shown that Bcl-2 protein expression is directly related to the grade of cervical intraepithelial neoplasia [27]. This is however in contrast to results in another study where it was demonstrated that Bcl-2 protein expression in neoplastic cervical tissue is significantly decreased in comparison to the normal cervical tissue [28].

## Conclusions

In summary, we have demonstrated that in cervical cancer cells, Bcl-2 is up-regulated as a potential means of providing resistance against cisplatin treatment. Silencing of Bcl-2 is able to greatly improve cisplatin sensitivity in both cell lines, particularly in aggressive CaSki cells. Therefore, the in vitro, as well as the ex vivo data strongly suggest that Bcl-2 is a promising therapeutic target for the treatment of cervical cancer. Here we show that it is possible to utilise lower doses of cisplatin while addressing cervical cancer resistance.
